# Oral Nano-Delivery of Crotoxin Modulates Experimental Ulcerative Colitis in a Mouse Model of Maximum Acute Inflammatory Response

**DOI:** 10.3390/ijms27010185

**Published:** 2025-12-24

**Authors:** Raquel Guedes de Oliveira Brito, Fernanda Narangeira de Araujo Neves, Larissa Ferreira de Almeida, Bruna Cristina Favoretto, Wafa Hanna Koury Cabrera, Nancy Starobinas, Jamile Macedo Garcia, Natália Coelho Couto de Azevedo Fernandes, José Luiz de Souza Lopes, Marcia Carvalho de Abreu Fantini, Pedro Leonidas Oseliero Filho, Olga Martinez Ibañez, Osvaldo Augusto Sant’Anna, Solange Massa, Orlando Garcia Ribeiro

**Affiliations:** 1 Immunogenetics Laboratory, Butantan Institute, São Paulo 05503-900, Brazil; raquel.brito@unesp.br (R.G.d.O.B.); nandanarangeira@hotmail.com (F.N.d.A.N.); bruna.favoreto@fundacaobutantan.org.br (B.C.F.); nancy.starobinas@butantan.gov.br (N.S.); ocmibanez@gmail.com (O.M.I.); solange.massa@butantan.gov.br (S.M.); 2 Physics Institute, University of São Paulo, São Paulo 05508-220, Brazilmfantini@if.usp.br (M.C.d.A.F.); pedroleonidasoseliero@hotmail.com (P.L.O.F.); 3 Pathological Anatomy Center, Adolfo Lutz Institute, São Paulo 01246-902, Brazil; jamile.garcia@usp.br (J.M.G.); nccafernandes@yahoo.com.br (N.C.C.d.A.F.); 4 Department of Physics, FFCLRP, University of São Paulo, Ribeirão Preto 14040-900, Brazil; zeluiz@if.usp.br; 5 Materials Innovation Factory, University of Liverpool, Liverpool L69 7ZX, UK; 6 Immunochemistry Laboratory, Butantan Institute, São Paulo 05503-900, Brazil; oasantanna@butantan.gov.br

**Keywords:** DSS, silica nanoparticles, inflammation, ulcerative colitis, crotoxin, mice

## Abstract

The incorporation of drugs into nanostructured silica has proven to be an effective strategy for delaying drug release, protecting against enzymatic degradation, and enhancing therapeutic efficacy. Specifically, crotoxin, a component derived from the venom of *Crotalus durissus terrificus*, exhibits notable analgesic and immunomodulatory properties. Previous studies have demonstrated that encapsulating crotoxin within SBA-15 nanostructured mesoporous silica not only reduces its toxicity and enhances its analgesic effects but also enables effective oral administration. Given its promising efficacy and the expanding interest in its application across various experimental models and potential therapeutic uses, this study aimed to conduct a detailed analysis of the physicochemical properties of crotoxin when incorporated into SBA-15 silica. Following characterization, the crotoxin–SBA-15 complex was orally administered to mice in an experimental model of ulcerative colitis (UC). The most widely adopted experimental model for studying UC involves the administration of dextran sodium sulfate (DSS) in drinking water to induce colonic inflammation in susceptible animals. In this study, we hypothesized that crotoxin incorporated into ordered mesoporous silica (SBA-15) could modulate DSS-induced UC. Crotoxin was successfully incorporated into SBA-15 and administered orally, as its physicochemical properties supported this route of delivery. Mice received the crotoxin–SBA-15 complex either at the onset of UC induction or on days 1 and 4 after DSS exposure. Seven days after the start of DSS administration, we observed a substantial reduction (approximately 50%) in Disease Activity Index (DAI) scores, accompanied by marked improvements in the histopathological features of the colon. These findings indicate for the first time that crotoxin incorporated into SBA-15 exhibits significant therapeutic potential in the treatment of experimentally induced ulcerative colitis.

## 1. Introduction

Mesoporous silica nanoparticles (MSNs) have been widely used in biomedical research due to their unique morphological features and ease of functionalization. These properties enable the loading of therapeutic agents, including small molecules, peptides, proteins, and genes, through electrostatic interactions or chemical bonding. The synthesis conditions of mesoporous silica materials can be precisely controlled to optimize their performance while minimizing toxicity. Materials in the SBA series feature a silica-based framework with a highly ordered mesoporous structure, adjustable pore sizes, high specific surface area, and excellent thermal stability. Among the various SBA materials, only SBA-15 and SBA-16 are commonly used in biomedical applications. Notably, SBA-15 is the most frequently selected carrier for large biomolecules due to its large surface area and pore diameter. Incorporating proteins into mesoporous silica enhances their stability, reduces susceptibility to degradation, and improves their therapeutic efficacy in experimental disease models [1].

Crotoxin (CTX), a protein extracted from the venom of the South American rattlesnake *Crotalus durissus terrificus* has been studied for its analgesic, anti-inflammatory, antitumor, and muscle-paralyzing activities. The venom exhibits neurotoxic, myotoxic, and coagulant effects [2], with these toxic actions being primarily attributed to crotoxin, the main component of the venom, which limits its direct medicinal use. Crotoxin has a molecular weight of approximately 24 kDa [3,4] and is composed of two subunits: a larger, basic subunit rich in lysine and arginine residues, known as crotapotin (CA), and a smaller, strongly acidic subunit with phospholipase A_2_ activity, known as CB [5]. Previous studies have shown that the toxicity of crotoxin can be reduced and its therapeutic effects enhanced when it is encapsulated in nanostructured SBA-15 silica. Oral administration of the complex reduced neuropathic pain, a chronic condition experimentally induced in mice by sciatic nerve injury. A decrease in the activation of central nervous system cells, such as astrocytes and microglia involved in inflammatory responses, was also observed [5,6].

Some authors have investigated the effects of free crotoxin on intestinal inflammation and observed that intraperitoneal (i.p.) administration of crotoxin reduced the classical symptoms of ulcerative colitis induced by 2,4,6-trinitrobenzene sulfonic acid (TNBS) in mice [7]. Ulcerative colitis (UC) is a chronic, relapsing form of inflammatory bowel disease (IBD) characterized by inflammation of the mucosal lining of the large intestine, primarily affecting the distal colon and rectum. Both genetic and environmental factors play crucial roles in the development of UC. Among environmental influences, industrialization and the westernization of lifestyles characterized by increased consumption of processed and high-fat foods, reduced intake of dietary fiber, greater use of antibiotics and medications, more sedentary lifestyles, and higher hygiene standards (which may impact immune system development) are particularly significant [5,8]. These factors can influence the gut microbiota and disrupt the epithelial barrier, leading to abnormal mucosal immune responses and inflammation that contribute to the development of UC. These responses are characterized by the production of pro-inflammatory cytokines, activation of inflammatory cells, and impaired immunoregulation. As a result, the mucosa becomes damaged, causing symptoms such as diarrhea, rectal bleeding, weight loss, abdominal pain, and other less severe manifestations [9,10,11].

Different experimental protocols can reproduce the pathophysiology of UC, with chemical induction using dextran sulfate sodium (DSS) dissolved in drinking water being one of the most commonly used methods [12]. DSS was initially used in hamsters in 1985 [13] and later in mice in 1990 by Ohkusa’s group [14], successfully reproducing a model similar to human UC. The symptoms are caused by the direct action of DSS on the epithelial cell barrier, leading to the translocation of gut microbiota from the intestinal lumen into the mucus layers. This triggers inflammatory responses characterized by cellular infiltration, mucin depletion, cryptitis, and the formation of crypt abscesses [15,16]. Due to its chronic nature, associated risks, and limited therapeutic options, ulcerative colitis (UC) significantly impacts patients’ quality of life. Ulcerative colitis is significantly more common in Western countries (Europe, North America, and Oceania), with incidence rates frequently exceeding 10 cases per 100,000 people per year. In developing countries, the rates are lower, ranging from approximately 0.5 to 6 cases per 100,000 people per year. The estimated global number of individuals living with ulcerative colitis is approximately 6 million, according to recent systematic reviews and data from the Global Burden of Disease (GBD) study (2017–2023) [17,18]. These indices highlight the growing importance of studying the disease [19,20]. Available therapies include palliative treatments, such as anti-inflammatory drugs, immunosuppressants, and biological agents that target specific components of the immune response. In more severe cases, disease progression may necessitate partial or total surgical removal of the colon [21,22,23]. Considering the characteristics of crotoxin and the potentiation of its effects when incorporated into SBA-15 mesoporous silica nanoparticles (CTX-SBA-15), this study presents, for the first time, a detailed biophysical characterization of crotoxin’s structure within a silica matrix. We then analyzed the effect of oral administration of the CTX-SBA-15 complex on the progression of DSS-induced ulcerative colitis using a susceptible mouse model.

The synthesis conditions were precisely controlled, ensuring that crotoxin in the complex remained stable. The CTX-SBA-15 complex exhibited a protective effect on the progression of ulcerative colitis.

## 2. Results

### 2.1. Analysis of Crotoxin Incorporated into SBA-15 Mesoporous Silica Nanoparticles

**Figure 1:**
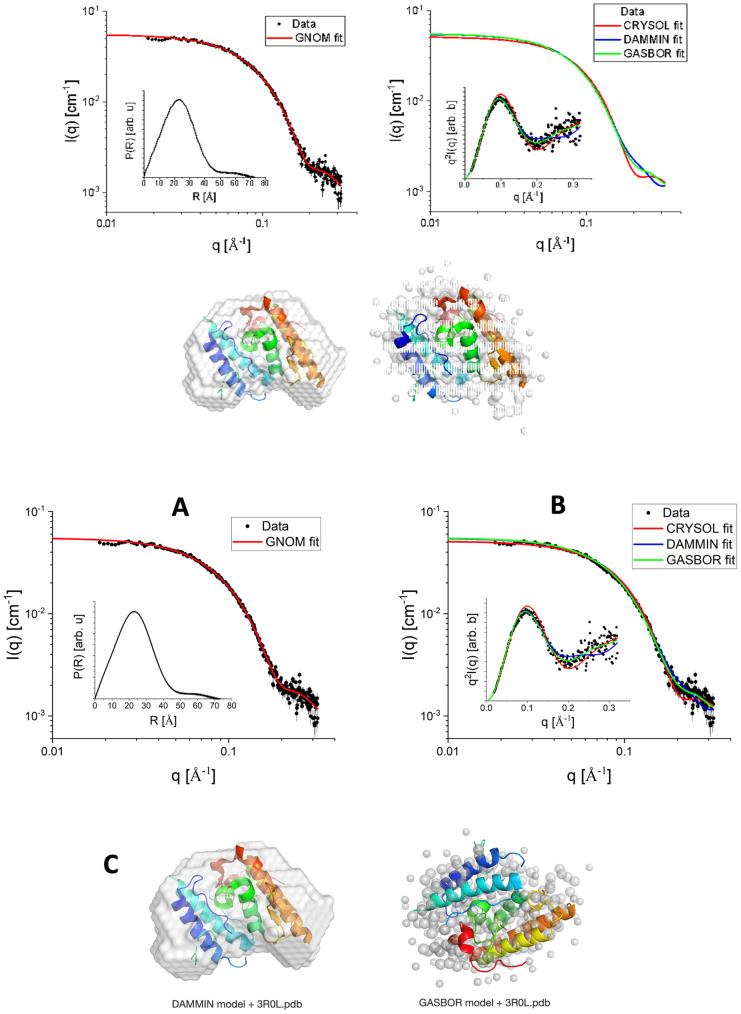
SAXS data (black filled circles) fitted with the IFT method using GNOM software (red continuous line). Inset: P(R) function obtained from the IFT fit (**A**). SAXS data were modeled using CRYSOL (PDBID 3R0L), DAMMIN, and GASBOR (red, blue, and green continuous lines, respectively). Inset: Kratky plot of experimental data and fittings (**B**). Comparison between the bead models obtained from DAMMIN and GASBOR with the PDBID 3R0L (**C**).

Figure 1A shows the SAXS experimental data (black-filled circles) of CTX in a PBS buffer. We first proceeded with the Indirect Fourier Transform to extract structural information from the curve, which brings information in the real space in a free modeling approach through the so-called pair-distance distribution function, P(R). The software GNOM version E 4.2 was used for this purpose [24]. From the satisfactory IFT fitting (red continuous line in Figure 1A), P(R) was obtained (inset of Figure 1A), which suggests the existence of particles with globular shape, possibly with flexible parts, having a maximum length (where P(R)~0) of approximately 70 Å. The hypothesis about the flexibility along the globular shape is corroborated when the data is visualized in a Kratky plot, i.e., I(q)xq^2^ versus q (inset of Figure 1B). Additionally, the maximum length of the particles is smaller than the mean mesopore size of the SBA-15, which reinforces the presence of CTX inside the mesopores.

From the IFT method, it is also possible to evaluate the protein radius of gyration, RG = (17.93 ± 0.06) Å, which is slightly smaller compared to the one reported in the literature [25], and the forward scattering, I(0) = (0.053 ± 0.002) cm^−1^, related to the protein molecular weight, M_W_ (in kDa), by [26]:

(1) $$M_{W}\;=\;\frac{I\left(0\right)\cdot N_{A}}{c\cdot {\left(\Delta \rho_{m}\right)}^{2}\;}\;\;$$

where *N_A_*, *c*, and Δ*ρ_m_* are Avogadro’s number, the concentration of the protein (in mg mL^−1^), and the excess scattering length density per unit mass (in cm g^−1^), respectively. A good approximation of Δ*ρ_m_* for proteins is 2 × 10^10^ cm g^−1^. Using *c* = 3.44 mg mL^−1^, obtained from UV-Vis spectroscopy measurements, we obtained *M_W_* ≈ 23.2 kDa, close to the theoretical value calculated from the PDBID 3R0L, indicating a quite-monodisperse sample.

Considering the high-resolution model 3R0L for CTX, one can compare the theoretically expected intensity with the obtained SAXS experimental data, for instance, using the CRYSOL program [27]. Proceeding in this way, we obtained a satisfactory fitting (Figure 1B, red continuous line), suggesting that the tertiary structure of CTX (overall shape and size) observed in its crystal form is also kept in solution, as well as the secondary structure, already observed in CD results (Figure 3A). This fact is corroborated by the good agreement between the high-resolution model of CTX and the ab initio bead model obtained from DAMMIN [28,29] programs, the former one using the amount of CTX residues as a constraint. The SUPALM program [30] was used to superimpose one 3D structure onto another, and the visual comparisons are shown in Figure 1.

**Figure 2:**
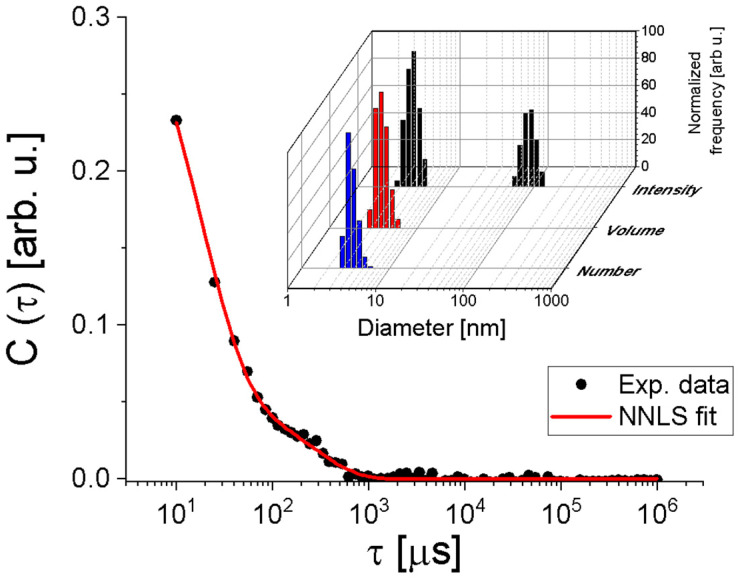
Autocorrelation function (filled circles) fitted by the NNLS method (continuous line). Inset: Hydrodynamic diameter distributions of the peptide weighted by number, volume, and intensity.

DLS was performed to check the presence of possible protein aggregates in aqueous dispersion, and the autocorrelation function, C(τ), is shown in Figure 2 (filled circles). By using the Non-Negatively constrained Least Squares (NNLS) method [31] to fit the curve C(τ) satisfactorily (Figure 2, red continuous line), the histograms of hydrodynamic diameter per intensity, per volume, and per number of particles were obtained (Figure 2, inset). Despite the presence of larger particles with a diameter of (91 ± 28) nm, they are significantly less numerous, as indicated by the histograms of diameter per volume and per number. Thus, most particles have a diameter of (4.3 ± 0.2) nm, corroborating that the samples are quite monodisperse and in complete agreement with SAXS analyses.

Notably, the obtained autocorrelation function is slightly different from the curve shown in Fernandes’ study in 2017 [25]. This could be because the authors did not use filtration before the measurements. Therefore, larger aggregates could meaningfully alter the measured data, as is known in any DLS experiment, even if these particles are not as numerous as the smaller ones. To check if DLS data is also consistent with the high-resolution model 3R0L of CTX, we used the HYDROPO software, version 10 [32], which allows the calculation of several hydrodynamic properties of rigid macromolecules, such as globular proteins, from their atomic-level structure. Using this, the predicted hydrodynamic diameter of CTX at the experimental conditions used in this investigation is ~4.6 nm, in agreement with the value obtained by DLS analysis. Furthermore, the estimated radius of gyration and the longest CTX length are ~17 Å and ~63 Å, respectively, in agreement with the SAXS results.

**Figure 3:**
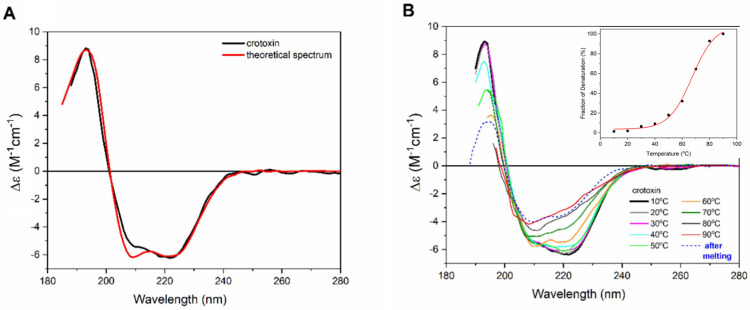
(**A**) Crotoxin CD spectra in PBS (black) and obtained from PDBID 3R0L (red). (**B**) Effect of temperature on the secondary structure of crotoxin. Crotoxin CD spectra, taken from 10 °C (black) to 90 °C (red). Intermediate temperatures are labeled in 10 °C steps, and the spectrum of the protein cooled back to 10 °C after treatment is dashed blue. Inset: Thermal melting curve for crotoxin, presenting Tm value of ~67 °C.

The CD spectra of crotoxin in PBS solution (Figure 3A and Supplementary Figure S1) show two minima in the 222 and 208 nm regions, and a positive maximum at 192 nm, which are characteristic of the alpha helix content of this protein [33].

Estimations of the secondary structure content based on this CD spectrum yielded values of ~49% α-helix, 8% β-strands, and 43% others, which are in close agreement with the protein crystal structure deposited on PDBID 3R0L (45% helix, 3% β-sheet, and 52% others). The deconvolution of the CD spectra of crotoxin was performed using the Dichroweb server with the SP175t [34] database and the ContinLL software Version 2. The increase in temperature was seen to cause severe conformational changes in the crotoxin structure (Figure 3B). Crotoxin remained thermally stable within the 10–40 °C interval, preserving its native CD spectrum profile unchanged up to 50 C. However, after crossing this point into higher temperatures (60–90 °C), significant conformational changes (such as a reduction in the 222 nm band, assumed to reflect a loss of helical content) were observed. The melting temperature (Tm) estimated for the thermal denaturation of crotoxin in PBS was ~67 °C. After cooling the sample back to 10 °C, the native CD spectrum of crotoxin was not reassumed, suggesting the protein has assumed a more unfolded/disordered state due to its thermal denaturation assay as an irreversible process.

**Figure 4:**
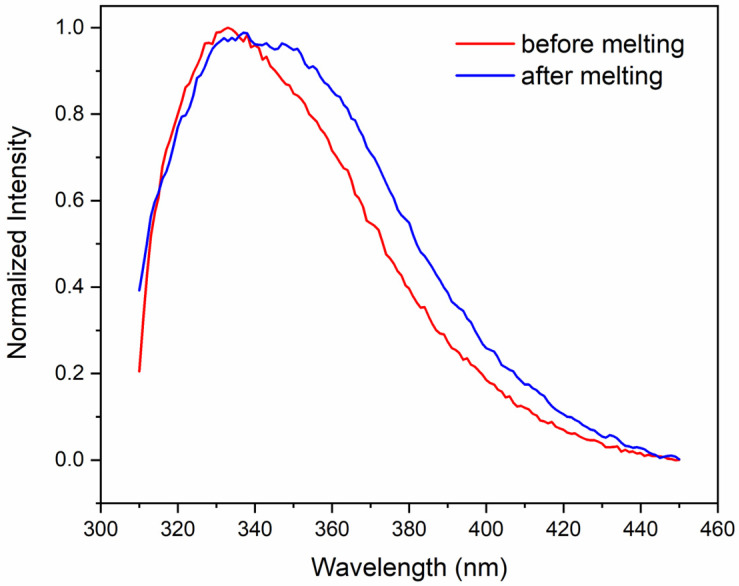
Fluorescence spectrum of crotoxin in PBS at 25 °C (red) and after thermal melting treatment (blue).

The emission spectrum of the Trp residues of crotoxin in PBS (Figure 4) shows a maximum at 333 nm, indicating the preservation of the aromatic residues from exposure to the aqueous environment, as is typically observed in globular proteins [35]. However, after heating the protein to 90 °C, the maximum emission shifted to ~345 nm, indicating a higher exposure of aromatic residues to the aqueous solvent following thermal treatment. In agreement with that, a reduction in the polarization values of crotoxin in PBS from ~0.17 to ~0.066 was also observed after thermal melting treatment, indicating the Trp residues assume a faster rotation in solution due to their higher local mobility.

Overall, the results demonstrate the efficiency of crotoxin incorporation into mesoporous silica. The analyses indicate that this process does not alter the secondary or tertiary structures of crotoxin in solution. The preservation of aromatic residues and the observed thermostability of the complex further support the structural integrity of the encapsulated crotoxin.

### 2.2. Crotoxin Incorporated into SBA-15 Mesoporous Silica Nanoparticles (CTX-SBA-15) Modulates DSS-Induced Colitis

We used crotoxin, incorporated into SBA-15 (CTX-SBA-15), as an anti-inflammatory agent to modulate DSS-induced intestinal inflammation. Behavioral observations confirmed that there was no toxicity or morbidity associated with the orally administered CTX-SBA-15 complex at various concentrations, ranging from 31.25 to 250 μg/kg body weight (Supplementary Figure S2). We also evaluated its release from SBA-15, mimicking the gastrointestinal tract environment in vitro (Supplementary Figure S3).

We divided the AIRmax mice into seven groups, as outlined in Section 4.

**Figure 5:**
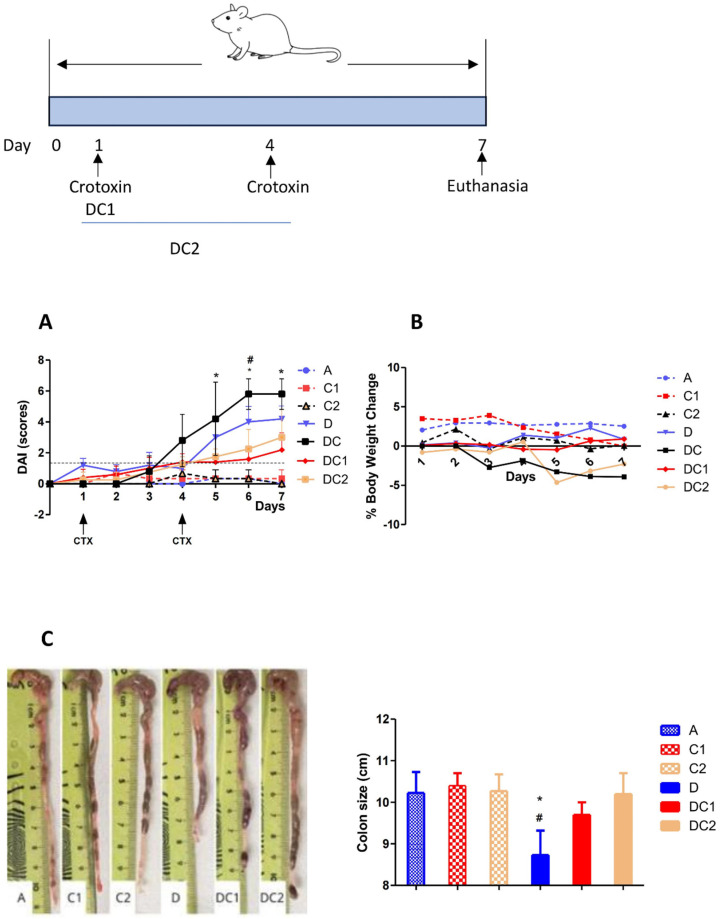
Disease Activity Index (DAI) (**A**), Body weight change (**B**), and Colon size (**C**). The group’s description is A-Negative control: intake of water only; D-Positive control: DSS 2.5% intake; C1-Control CTX single dose 125 μg/Kg; C2-Control CTX two doses 125 μg/Kg; DC-2.5% DSS + CTX single dose without SBA15; DC1-DSS 2.5% + CTX single dose 125 μg/Kg; DC2-DSS 2.5% + CTX two doses 125 μg/Kg. In all groups, the CTX was incorporated into SBA-15. Values expressed as mean ± SEM, of 5 animals/group comparisons between DSS-treated and DC1 groups (*) or between DSS and DC2 mice (#) using ANOVA, *p* < 0.05.

Figure 5 shows that mice treated with CTX-SBA-15 alone or with water exhibited low DAI scores, while groups that received DSS, with or without CTX-SBA-15, showed elevated scores. However, administration of CTX-SBA-15, whether as a single or double dose, resulted in a significant reduction in DAI. This reduction was particularly noticeable starting on the fifth day following the initiation of DSS treatment, coinciding with the peak scores in the DSS-only group. In the group that received two gavage doses of CTX-SBA-15, a noticeable decrease in DAI was observed starting on day 6. It is worth noting that crotoxin at 125 µg/kg, without SBA-15, does not exhibit any UC-modulatory effect as measured by DAI. Treatment with DSS resulted in a reduction in colon length, a key indicator of disease progression in experimental colitis. This effect was completely reversed in animals treated with DSS and one or two doses of CTX-SBA-15.

**Figure 6:**
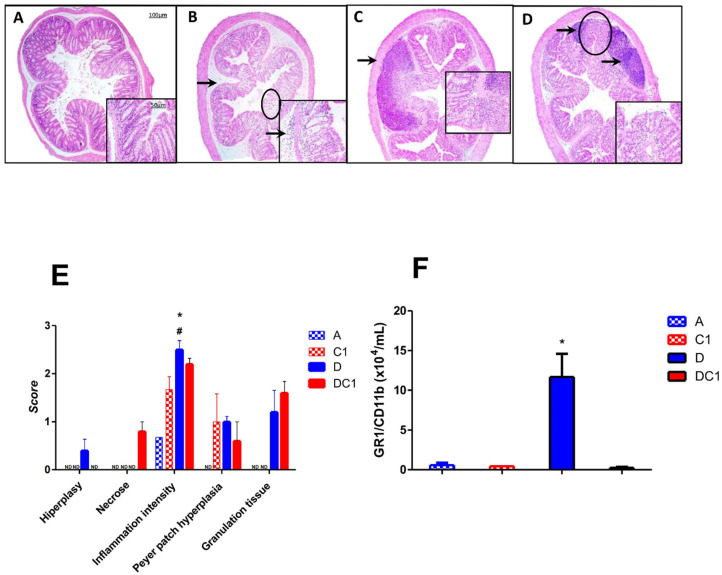
Histopathological analysis. Representative photomicrographs (Magnification of 40× and inset 200×) from AIRmax mice. (**A**)—typical colonic tissue with access to pure water, (**B**)—DSS 2.5% ad libitum indicating submucosa edema associated with inflammatory infiltrate (arrow) and area of glandular cells loss (circle), (**C**,**D**)—DSS 2.5% and SBA-15-crotoxin 125 µg/Kg bw at the first day (**C**) or at first and fourth days (**D**) indicating Peyer’s patch hyperplasia (arrows) and fibroplasia with mild inflammatory infiltrate (circle in (**D**)), lower loss of glandular cells compared to the DSS group. Notably, there is no edema, with preservation of the epithelium and villous architecture. (**E**)—Histopathological characteristics are measured by scores of hyperplasia, necrosis, inflammation intensity, and Peyer patch hyperplasia alterations at the seventh day post-treatment. (**F**)—GR1^+^CD11b^+^ cells (neutrophils) were detected in the colon by flow cytometry on the seventh day post-treatment. The values shown are the means ± SEM of 5 animals per group. * Difference between DSS-treated and control mice, # Difference between DSS and DC-treated mice, by ANOVA, *p* < 0.05. ND = not detected.

Histological analysis revealed that the control groups, which received only water or CTX-SBA-15 (1 or 2 doses), preserved normal colonic epithelial architecture, characterized by visible villi and distinct cell types within the crypts. In contrast, the DSS-treated group displayed epithelial disruption and a marked inflammatory response in the submucosal layer, characterized by neutrophilic infiltration. In the group treated with DSS and CTX-SBA-15, a small but significant reduction in inflammation severity was observed compared with the DSS-alone group (Figure 6 and Supplementary Figure S4).

## 3. Discussion

In this study, we focused on the promising potential of crotoxin as an orally administered modulatory agent for the treatment of experimental ulcerative colitis. To ensure its stability and bioavailability, crotoxin was incorporated into nanostructured silica, a crucial step that protects the molecule during its passage through the digestive tract and enables its delivery to the colonic epithelium [36].

Nanostructured silicas are silicon oxide [SiO_2_] particles with a highly organized mesopore structure that, due to their physicochemical properties, have the potential for use in different areas and biological applications [6,37]. These materials can interact with atoms, ions, and molecules not only on their surfaces but also within their interiors. As described, SBA-15 silica has a hexagonal structure with highly ordered and interconnected pores, relatively thick walls (up to 6 nm), pore size around 10 nm, pore volume greater than 1 cm^3^g^−1^, surface area above 800 m^2^g^−1^, a large number of silanol groups, which provides high adsorption/incorporation capacity for various organic, inorganic and biological species, in addition to notable thermal, hydrothermal and mechanical stability [38]. In the present study, we employed multiple techniques such as Small-Angle X-ray Scattering (SAXS), Circular Dichroism (CD), fluorescence spectroscopy, UV-Vis spectrophotometry, and Dynamic Light Scattering (DLS) to analyze various aspects of the nanostructured silica-crotoxin (CTX-SBA-15) complex. These included the structural organization of the material, secondary structure, and conformational changes of the protein upon incorporation into silica, alterations in protein folding, ligand binding, interactions with other molecules, or aggregation, and the quantification of crotoxin loading into SBA-15. Collectively, these techniques provided a comprehensive understanding of the CTX-SBA-15 interaction, demonstrating that the structural, conformational, chemical, and functional properties of crotoxin were preserved within the complex.

Toxins derived from venomous animals such as snakes, scorpions, and spiders have contributed significantly to human health, for example, the development of the antihypertensive drug Captopril^®^, which originated from studies on *Bothrops jararaca* venom [39,40,41]. Crotoxin, a component of the venom of snakes from the genus *Crotalus*, exhibits antinociceptive and immunomodulatory effects in vivo, making it a promising candidate for pharmacological intervention in inflammatory diseases [4,7,42]. However, the therapeutic application of such toxins is often limited by their inherent toxicity. To mitigate these effects and enhance oral bioavailability, we encapsulated crotoxin in mesoporous SBA-15 silica [43].

As an experimental model, we utilized a line of mice with high inflammatory capacity, derived from heterogeneous populations through selective breeding, to induce a high acute inflammatory response with polyacrylamide particles [44]. These mice develop significant DSS-induced intestinal inflammation that closely mimics human ulcerative colitis [45].

In our in vivo experiments, we administered varying doses of CTX-SBA-15 to assess its potential toxicity upon oral delivery. Based on behavioral observations, no detectable toxic effects or mortality were observed at the tested doses, supporting its safety in mice. One hypothesis is that SBA-15 reduces the toxic effects of crotoxin when encapsulated [46], which is consistent with our findings. SBA-15 likely functions as both a carrier and a protective matrix, shielding crotoxin throughout the gastrointestinal tract and facilitating its delivery to the therapeutic target [46,47].

We used therapeutic concentrations of crotoxin, similar to those reported in studies by Sant’Anna [6,46]. Previous research by Almeida (2015) demonstrated that free crotoxin, when administered intraperitoneally, can modulate acute intestinal inflammation induced by rectal administration of TNBS in mice [7]. The results of our current experiments are consistent with these findings, particularly in the group treated with DSS and a single oral dose of CTX-SBA-15. In this group, we observed a reduction in the DAI, primarily due to improved clinical signs, as well as the absence of colon shortening, a decrease in inflammatory infiltrate, and preservation of the colonic epithelium, compared to the control DSS-treated group.

## 4. Materials and Methods

### 4.1. Crotoxin Purification

Crotoxin was purified from *Crotalus durissus terrificus* venom using the method described by Bon et al. (1989) [48]. Briefly, aliquots of venom diluted in phosphate buffer (50 mM, pH 7) were subjected to anion exchange chromatography on a MONO-Q HR 5/5 column in an Akta-FPLC system (GE Healthcare, São Paulo, Brazil). The proteins adsorbed to the resin were eluted using a linear gradient from 0 to 1 M NaCl, buffered with 50 mM phosphate. The fractions corresponding to CTX were pooled and dialyzed against PBS, and the protein concentration was determined by the BCA method. For SDS-page analysis, 20 µL (15 µg) of venom or CTX were loaded in 12% SDS-PAGE under reducing conditions (2% β-mercaptoethanol). The gels were Coomassie Brilliant Blue-stained (Supplementary Figure S5).

### 4.2. Preparation of the Complex Crotoxin and SBA-15 Mesoporous Silica (CTX-SBA-15)

Crotoxin was diluted in phosphate-buffered saline (PBS, pH 7.4) and gradually incorporated into SBA-15 mesoporous silica at a 1:10 ratio (CTX:SBA-15), corresponding to 125 μg/kg body weight. The mixture was maintained at 2–8 °C for 24 h with occasional stirring. The profile of crotoxin liberation from the complex in different pHs is presented in Supplementary Figure S3.

### 4.3. CTX-SBA-15 Characterization

The crotoxin used in this study was characterized by Small-Angle X-ray Scattering (SAXS), Circular Dichroism (CD), fluorescence spectroscopy, UV-Vis spectrophotometry, and Dynamical Light Scattering (DLS).

SAXS experiment was performed on a Nanostar (Bruker, Mannheim, Germany) instrument equipped with a microfocus Genix 3D system (Xenocs, Grenoble, France) and a Pilatus 300k (Dectris, Lund, Sweden) detector. The sample-to-detector distance was ~667 mm, which provided an effective range of the modulus of the transfer moment vector, q = 4πsin(θ)/λ (where 2θ is the scattering angle and λ = 1.5418 Å is the X-ray wavelength), from 0.018 to 0.30 Å^−1^. We measured the CTX sample (at 5 mg/mL) and PBS buffer in the same reusable quartz capillary with a 1.5 mm diameter mounted on stainless steel cases. Data statistics were satisfactory at this concentration, and no interparticle interaction was observed. Data treatment, including azimuthal integration, background subtraction, and absolute scale normalization, was performed using the SUPERSAXS software suite, version 02-06-2022 [49].

DLS measurement was performed on a Brookhaven DM-5000 particle-size analyzer (Brookhaven Instruments, Nashua, NH, USA) at room temperature using a wavelength of 635 nm. Data analysis was conducted using the BIC software version 3.0, provided with the machine. Before the measurements, the CTX sample was filtered using a 0.45 µm filter. Then, with the help of UV-Vis spectroscopy, the concentration was adjusted to 1 mg/mL (by adding PBS buffer) to mitigate the influence of any interparticle interaction on the diffusion coefficient of the particles and, consequently, on the obtained data.

UV-Vis spectrophotometry was performed on a NanoDrop 2000 (Thermo Fisher, Waltham, MA, USA) using 0.5 mg/mL CTX in PBS.

The CD spectra of crotoxin (0.1 mg/mL) in PBS were collected on a J-815 spectropolarimeter (Jasco, Tokyo, Japan) from 280 nm to 190 nm in 1 nm intervals, using a scan speed of 50 nm/min and a 1.0 mm pathlength quartz Suprasil (Hellma, Müllheim, Germany) cuvette at 25 °C. Thermal stability studies of crotoxin in PBS were performed over the temperature range of 10 to 90 °C in 10 °C increments, allowing 5 min of equilibration time at each temperature before measurement. Six repeat measurements were made at each temperature. CDToolX [50] software version 1.17, was used for data processing, which consisted of the average of the six scans of the sample, subtraction of the corresponding buffer baseline, smoothing with a Savitzky–Golay filter, and zeroing at the 263–270 region. Data was converted to delta epsilon (Δε) units using a mean residue weight of 112.9. The secondary structural content was analyzed using the Dichroweb server [51] with the SP175t database [34].

The emission spectra of the tryptophan (Trp) fluorescence spectroscopy residues in crotoxin (0.075 mg/mL) in PBS were collected on an ISS K2 spectrofluorometer (Champaign, IL, USA) with excitation performed at 295 nm, using 8 nm slits for both excitation and emission, in a 1 cm pathlength quartz cuvette Hellma, at 25 °C. Emission spectra were recorded over the wavelength range from 305 to 450 nm. The fluorescence polarization of crotoxin in PBS was measured with excitation at 280 and emission monitored at 340 nm. Fluorescence polarization (P) was determined as a function of the parallel ($I_{//}$) and the perpendicular (*I* ) fluorescence intensities, according to the equation [35]:

(2) $$P=\frac{I_{//}-I\;}{I_{//}+I\;}\;\;\;$$

### 4.4. Mice

We used 3-month-old male mice from the AIRmax mouse line [44], maintained in the animal facilities of the Immunogenetics Laboratory at the Butantan Institute (São Paulo, Brazil). The animals were housed individually in cages with wood shavings and kept on a 12 h light/dark cycle. The animal study protocol was approved by the Animal Ethics Committee of the Butantan Institute, under protocol code 6966270422, dated 6 March 2022.

### 4.5. Ulcerative Colitis Induction and CTX-SBA-15 Treatment

We organized the AIRmax mice into seven experimental groups with five mice per group:

A—Negative control: water and food ad libitum;

D—Positive control: 2.5% DSS (MP Biomedicals, LLC, Illkirch-Graffenstaden, France) in distilled drinking water for seven consecutive days and food ad libitum;

C1—CTX-SBA-15 control (single dose): a single oral dose (gavage) of CTX-SBA-15 at 125 μg/kg bw in 300 µL volume with water and food ad libitum;

C2—CTX-SBA-15 control (two doses): two oral doses (gavage) of CTX-SBA-15 at 125 μg/kg bw, administered four days apart, with water and food ad libitum;

DC—DSS + CTX: a single oral dose of CTX at 125 μg/kg bw 30 min before 2.5% DSS in drinking water for 7 days and food ad libitum;

DC1—DSS + CTX-SBA-15 (single dose): a single oral dose of CTX-SBA-15 at 125 μg/kg bw 30 min before 2.5% DSS in drinking water for 7 days and food ad libitum;

DC2—DSS + CTX-SBA-15 (two doses): two oral doses of CTX-SBA-15 at 125 μg/kg bw 30 min before and 4 days after 2.5% DSS in drinking water for 7 days and food ad libitum.

The animals were monitored for seven days using the Disease Activity Index (DAI).

At the end of the seven-day period, mice were euthanized by an overdose of ketamine (300 mg/Kg) and xylazine (30 mg/Kg) for colon collection and subsequent analysis. This experiment was independently repeated three times.

### 4.6. Disease Activity Index (DAI)

The Disease Activity Index (DAI) was evaluated based on changes in body weight, stool consistency, and the presence of blood in the feces or at the anus, as described in Table 1 [52,53]. Body weight was recorded before treatment initiation and daily for seven consecutive days following DSS administration. The index of diarrhea and rectal bleeding was also recorded daily. DAI scores were calculated by summing the individual scores for weight loss, stool consistency, and presence of blood and rectal bleeding, as outlined in Table 1. On day seven, animals were euthanized, and colon segments were collected. The colons were washed with saline and measured for length. The distal portion of each colon was then divided into three 1 cm segments for histological analysis.

### 4.7. Histological Analysis

Distal colon segments were removed, fixed in 10% paraformaldehyde for 24 h, and stored in 70% ethanol until further processing for histology. The samples were embedded in paraffin and sectioned at 5 µm for Hematoxylin and Eosin (H&E) staining, according to the standard protocol. For histopathological analysis, abnormal tissues were evaluated using the scoring system described by Erben et al. (2014) [54], which assesses the extent and severity of inflammatory cell infiltrates, epithelial alterations, and mucosal architectural changes.

### 4.8. FACS Analysis

Distal colon segments were incubated in digest solution (1 mg/mL collagenase and 1 U/mL DNase I in RPMI supplemented with 5% fetal calf serum, 1% L-glutamine, 1% penicillin-streptomycin, and 10 mM HEPES) for 40 min, at 37 °C. Resulting cell suspension was filtered in a 100 μm strainer, and purified using 35% Percoll. Following centrifugation at 400× *g*, the pellet was suspended in 1 mL of complete RPMI medium and counted in a Malassez hemocytometer chamber. Cell aliquots (100 μL at 1 × 10^5^ cells/mL) were labeled with specific rat antibodies against GR1 (clone RB6-8C5), and CD11b (clone M1/70). Phycoerythrin-labeled (PE)- or fluorescein isothiocyanate (FITC)-labeled mouse IgG2b (clone: A95-1) were used as control isotypes. To distinguish live cells from dead cells we used Fixable Viability Stains (FVS-Pacific Blue). All antibodies were purchased from BD Biosciences (Pharmigen, Franklin Lakes, NJ, USA). We recorded 50,000 events using a FACSCanto II flow cytometer (Becton Dickinson, Franklin Lakes, NJ, USA) and analyzed with FlowJo software 10.10.0 (Tree Star). The cell concentrations were calculated by multiplying the total number of viable cells obtained per mL (counted using Trypan blue exclusion) by the percent of the viable cells gated during flow cytometric analysis. Gating strategy is shown in Supplementary Figure S4.

### 4.9. Statistical Analysis

We used a one-way analysis of variance (ANOVA) to statistically determine significant differences between the mean values of the groups; *p* < 0.05 was considered significant. Data were analyzed using GraphPad Prism 4.0 software (GraphPad Software, San Diego, CA, USA).

## 5. Conclusions

Several advanced analytical techniques were used to assess the structure, stability, and physical properties of crotoxin and to verify that crotoxin was successfully incorporated into the mesoporous silica (SBA-15) structure. This configuration enables the time-controlled release of crotoxin, thereby reducing its toxicity while enhancing therapeutic efficacy. SBA-15-crotoxin demonstrated a significant modulatory effect on DSS-induced experimental ulcerative colitis, evidenced by improvements in the Disease Activity Index (DAI), macroscopic colon appearance, and histological features. Notably, treatment resulted in the preservation of the colonic epithelium and a reduction in inflammatory infiltrates. These initial findings suggest that SBA-15 serves as a protective and efficient delivery system, increasing crotoxin’s local bioavailability in the colon and effectively attenuating acute intestinal inflammation. Further studies are needed to elucidate the mechanisms and therapeutic role of the SBA-15–crotoxin complex in the prevention and/or treatment of inflammatory bowel diseases.

## Figures and Tables

**Table 1:** Disease activity index score parameters [53].

Stool Consistency	Bleeding	Weight Loss
0 = formed	0 = normal color stool	0 = no weight loss
1 = mild-soft	1 = brown color	1 = 5–10% weight loss
2 = very soft	2 = reddish color	2 = 11–15% weight loss
3 = watery stool	3 = bloody stool	3 = 16–20% weight loss
		4 = ≥20% weight loss

## Data Availability

The original contributions presented in this study are included in the article/Supplementary Material. Further inquiries can be directed to the corresponding author.

## Supplementary Materials

The following supporting information can be downloaded at: https://www.mdpi.com/article/10.3390/ijms27010185/s1.
