# Supplementary material for: Oral Nano-Delivery of Crotoxin Modulates Experimental Ulcerative Colitis in a Mouse Model of Maximum Acute Inflammatory Response
Source: Int J Mol Sci. 2025 Dec 24;27(1):185. doi: 10.3390/ijms27010185 (PMC12785686; doi:10.3390/ijms27010185)
Supplement: Supplementary file 1 [file ijms-27-00185-s001.zip › Supplementary Figure S1.pdf]

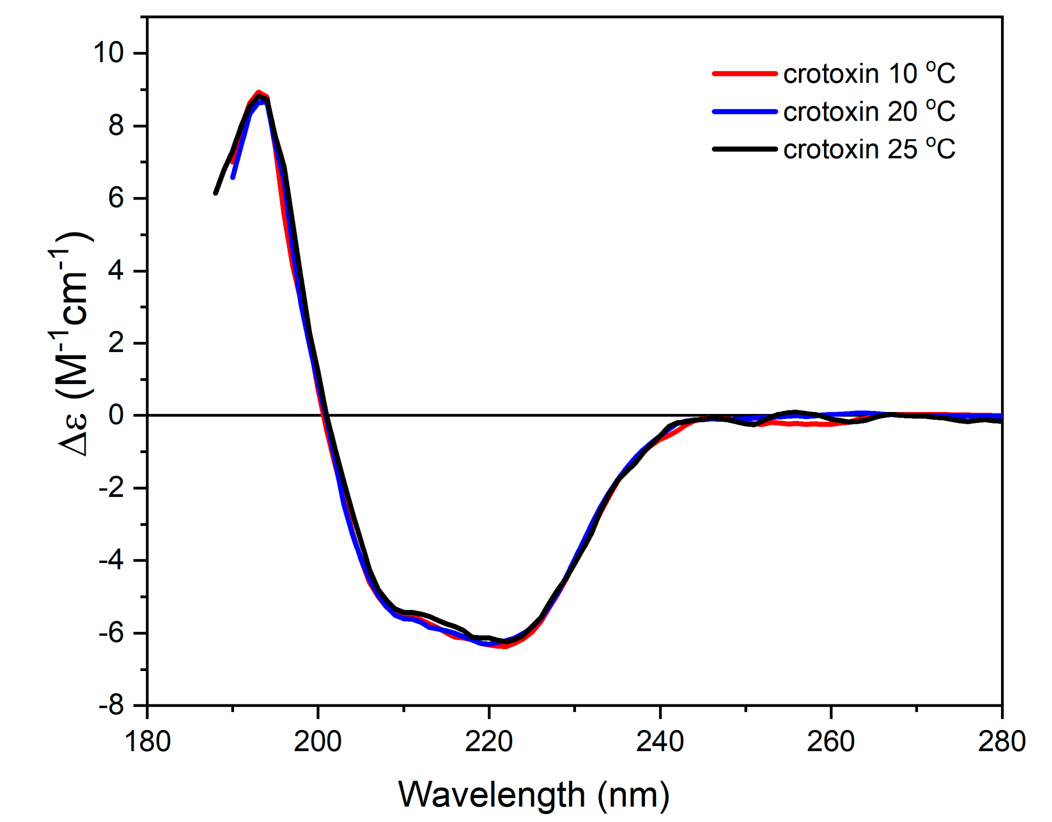

Supplementary Figure S1. CD spectra of crotoxin in PBS recorded at 10°C, 20°C, and 25°C. The nearly identical spectra indicate that the protein's secondary structure is maintained within this temperature range.
