# Supplementary material for: Oral Nano-Delivery of Crotoxin Modulates Experimental Ulcerative Colitis in a Mouse Model of Maximum Acute Inflammatory Response
Source: Int J Mol Sci. 2025 Dec 24;27(1):185. doi: 10.3390/ijms27010185 (PMC12785686; doi:10.3390/ijms27010185)
Supplement: Supplementary file 1 [file ijms-27-00185-s001.zip › Supplementary Figure S2.pdf]

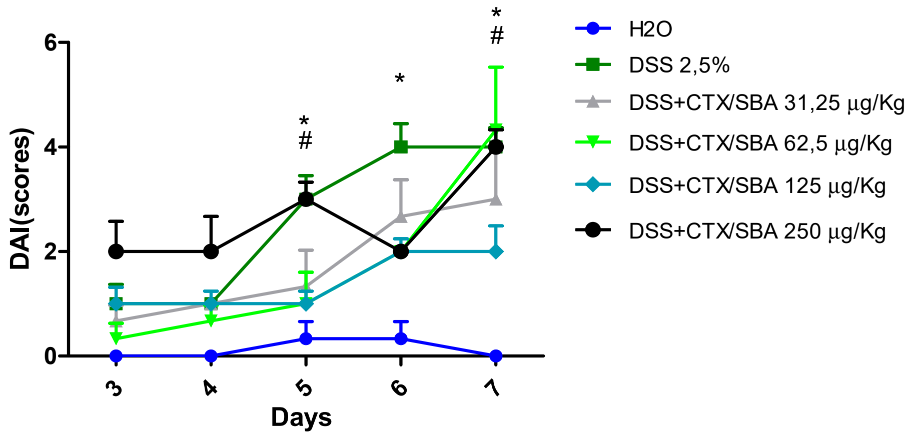

Supplementary Figure S2. Disease Activity Index (DAI) in response to oral administration (by gavage) of four doses of crotoxin incorporated into SBA-15. Data are presented as mean ± SE (n = 3). *p < 0.05 vs. DSS 2.5%; #p < 0.05 vs. CTX/SBA 125 µg/kg.
