# Supplementary material for: Oral Nano-Delivery of Crotoxin Modulates Experimental Ulcerative Colitis in a Mouse Model of Maximum Acute Inflammatory Response
Source: Int J Mol Sci. 2025 Dec 24;27(1):185. doi: 10.3390/ijms27010185 (PMC12785686; doi:10.3390/ijms27010185)
Supplement: Supplementary file 1 [file ijms-27-00185-s001.zip › Supplementary Figure S3.pdf]

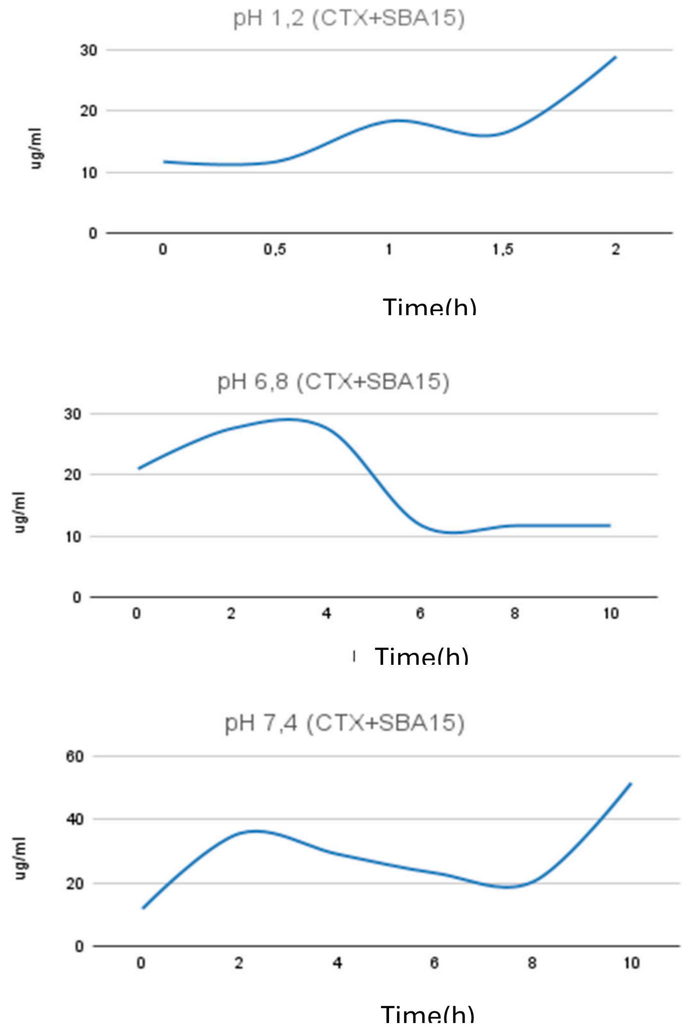

Supplementary Figure S3 - CTX Release Profile. We evaluated the release profile of CTX (250 µg/mL) from SBA-15 in vitro at physiological pH values (1.2, 6.8, and 7.4) over up to 10 hours.

- At pH 1.2 (Simulated Gastric Fluid): The release was relatively low and sustained. CTX showed an initial release of about 12% which gradually increased to approximately 30% after 10 hours.
- At pH 6.8 (Simulated Small Intestinal Fluid): A significant initial burst release was observed, reaching 20-30 % within the first 4 hours. Following this initial phase, the release rate slowed down considerably, resulting in only an additional 12 % release over the remaining 6 hours (from hour 4 to hour 10).
- At pH 7.4 (Simulated Colonic Fluid pH): The release profile displayed a bimodal curve. There was a moderate initial release in the early hours, followed by an accelerated release phase in the final hours (e.g., the last 2 hours), with the cumulative release ultimately reaching the highest value of about 40% at 10 hours.
