# Supplementary material for: Oral Nano-Delivery of Crotoxin Modulates Experimental Ulcerative Colitis in a Mouse Model of Maximum Acute Inflammatory Response
Source: Int J Mol Sci. 2025 Dec 24;27(1):185. doi: 10.3390/ijms27010185 (PMC12785686; doi:10.3390/ijms27010185)
Supplement: Supplementary file 1 [file ijms-27-00185-s001.zip › Supplementary Figure S4.pdf]

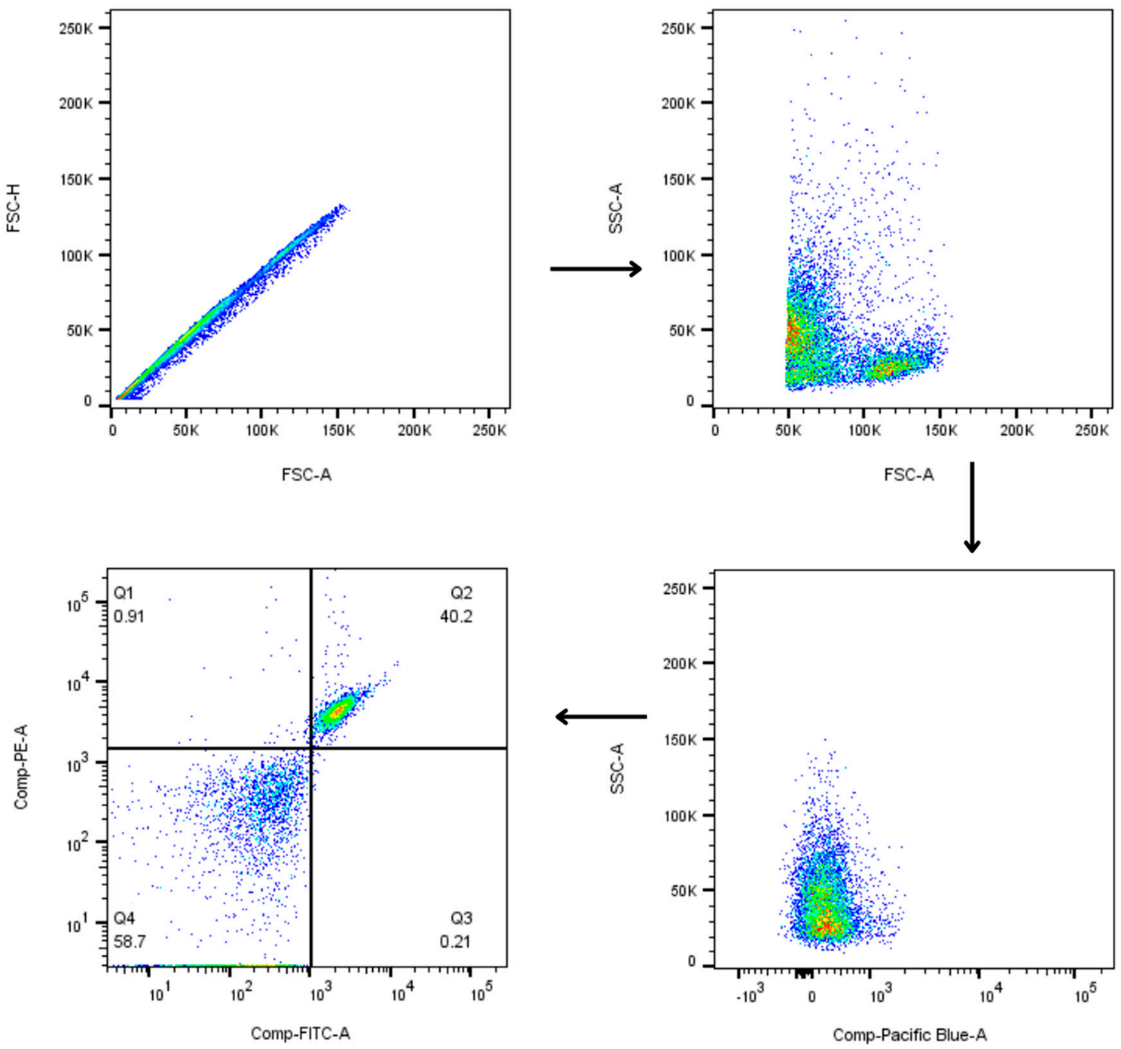

Supplementary Figure S4. Immunophenotyping analysis strategy of GR1/CD11b-labeled cells (Q2) on day 7 of the experiment (n = 3-5 mice/group). The panels refer, respectively, to singlet cells (A), the cells to be analyzed (B), live cells (FVS-Pacific Blue) (C), and the GR1/CD11b staining analysis (D).
