# Supplementary material for: Oral Nano-Delivery of Crotoxin Modulates Experimental Ulcerative Colitis in a Mouse Model of Maximum Acute Inflammatory Response
Source: Int J Mol Sci. 2025 Dec 24;27(1):185. doi: 10.3390/ijms27010185 (PMC12785686; doi:10.3390/ijms27010185)
Supplement: Supplementary file 1 [file ijms-27-00185-s001.zip › Supplementary Figure S5.pdf]

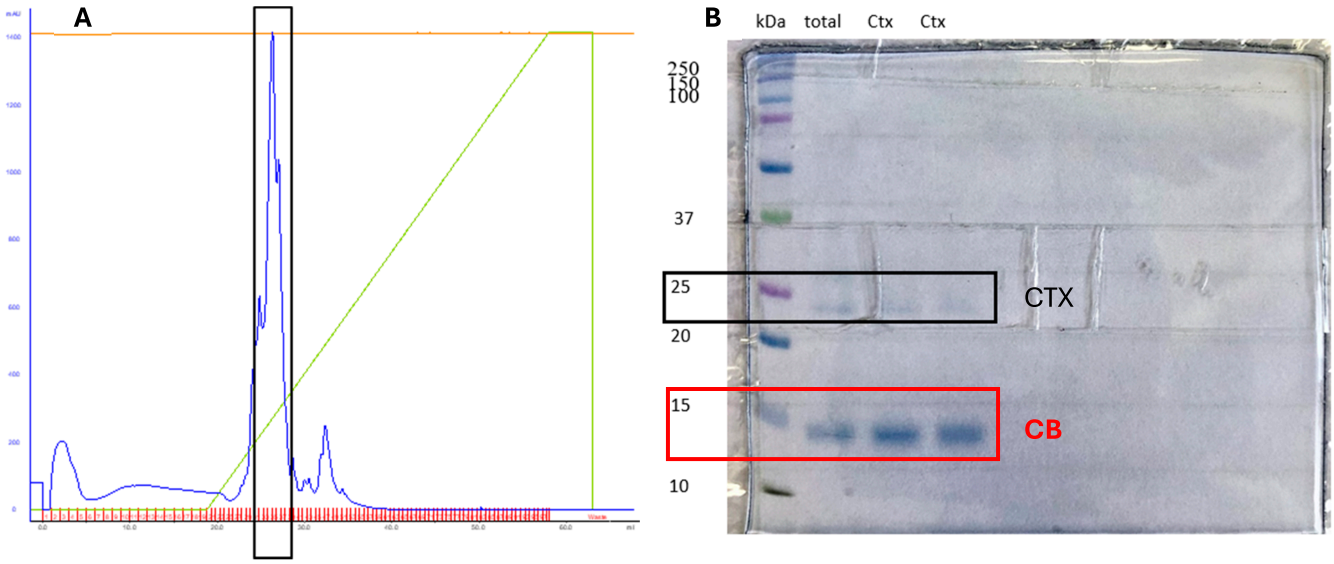

Supplementary Figure S5. (A) Anion exchange chromatography on a MONO-Q HR 5/5 column in an Akta-FPLC system (mAU x mL). The Crotoxin peak corresponds to the one highlighted in graph. **(B)** The SDS-PAGE confirms that the purified fraction contains two bands corresponding to crotoxin (black) and CB (red) fractions.

There is a dissociation in the CA and CB fractions, with the more intense fraction corresponding to the PLA2 fraction (CB). We can observe that the CB fraction (14 kDa) shows greater intensity than the band corresponding to purified crotoxin (24 kDa), while the CA band (9 kDa) may have come out of the gel. Because crotoxin is the major component of rattlesnake venom, it is not possible to clearly visualize other bands representing different proteins.
